# Intra-Arterial Prostaglandin E_1_ Infusion in Patients with Rest Pain: Short-Term Results

**DOI:** 10.1100/2012/803678

**Published:** 2012-03-12

**Authors:** A. Chatziioannou, A. Dalakidis, K. Katsenis, V. Koutoulidis, D. Mourikis

**Affiliations:** ^1^Department of Interventional Radiology, Aretaieion Hospital, University of Athens, 76 Vas. Sofias Avenue, 115 28 Athens, Greece; ^2^Department of Radiology, Aretaieion Hospital, 76 Vas. Sofias, 115 28 Athens, Greece; ^3^Department of Vascular Surgery, Aretaieion Hospital, Aretaieion Hospital, 76 Vas. Sofias Avenue, 115 28 Athens, Greece

## Abstract

*Purpose*. To present our results after short-term (1 month) intra-arterial infusion therapy of PGE_1_-alprostadil via a port system implanted in the ipsilateral external iliac artery (EIA) in patients with severe rest pain. *Methods*. Ten patients with severe rest pain were included. All patients showed extensive peripheral vascular disease below the knee. The tip of the catheter was introduced via a retrograde puncture in the ipsilateral external iliac artery (EIA). The patients received intraarterial infusion of PGE_1_, 20 mgr alprostadil daily, via the port catheter for 1 month. *Results*. Clinical success was evaluated according to subjective grading of pain (group A significant decrease, group B moderate decrease and group C no response). A significant decrease of rest pain was observed in 8 (group A, 80%) patients, a moderate decrease in 2 (Group B, 20%), whereas no patients demonstrated any significant response. Both patients of group B had Buergers' disease and continue to smoke during therapy. No peripheral thrombosis or clinical deterioration was noticed. *Conclusion*. Intraarterial infusion of PGE_1_ alprostadil on a daily basis, using a port catheter into the ipsilateral EIA, in selected patients with severe rest pain, seems to be very effective, without any serious complications.

## 1. Introduction

Prostaglandin E_1_ has been reported to benefit patients with significant peripheral vascular disease and limb threatening ischemia [1]. The routes of infusion may be either intravenous or intra-arterial [2–4]. Previously Strecker et al. [3] reported the use of an implantable port with its catheter placed mainly (9 out of 10 patients) towards the periphery of the leg for intra-arterial PGE_1_ infusion.

In the present study, we evaluate the same port in 10 patients with severe rest pain (Fontaine III), with its catheter placed in retrograde fashion away of the periphery of the leg, into the ipsilateral external iliac artery, after puncturing the common femoral artery of the same leg. The rationale of our study was to eliminate the risk of target vessel thrombosis since EIA has a larger diameter than peripheral arteries.

## 2. Materials and Methods

Ten patients (all men; age range: 34–71 yrs) with severe rest pain (Fontaine's stage III) were included into the study. Five of them had Buergers' disease. The rest had extensive peripheral atherosclerotic changes due to long standing diabetes.

All patients showed a very poor run-off with total occlusion of all three vessels slightly distally to trifurcation. Two patients with Buergers' disease had additional involvement of the popliteal artery. No patient was suitable for by-pass surgery because on selective arteriography no adequate distal vessels were identified. All patients suffered from significant rest pain, and one type of amputation could be speculated for them as the only potential treatment of choice.

Patients with significantly restricted external iliac artery inflow, unwilling to follow a daily hospital appointment, with significant alterations in blood coagulations measurements, heart failure, allergy to iodine contrast, PGE-1, or titanium port, were excluded from this study. Additionally, stage IV (ulcerations, osteomyelitis, or gangrene) patients were also excluded, since the effectiveness of the treatment was exclusively addressed for a short period in the subgroup of patients suffering from severe rest pain.

The catheters were placed in a retrograde fashion into the external iliac artery via puncturing of the ipsilateral common femoral artery. In our opinion, this change in location is an important parameter for elimination of the potential untowards effects. The port system (PIPS, Cook Europe) was implanted in the same manner as previously described [2, 3]. No systemic anticoagulation was administered.

After the implantation, PGE_1_ (20 mcg alprostadil, Pharmacia & UpJohn) diluted in 60 mL normal saline was infused daily over 60 min, via the port, on a outpatient basis. Subjective evaluation of the rest pain was determined at the end of each week. Brachial-tibial index was correlated before and at the completion of the treatment.

## 3. Results

The catheter port system could be successfully implanted in all 10 patients without any significant complications. All of the patients experienced decrease of the pain from the first week which continued through the following weeks. 8 patients (group A) experienced significant decrease of pain and 2 (group B) moderate decrease. The patients of group B had Buergers' disease with occlusion of the popliteal artery and no run-off peripherally.

No infection, migration, or leakage of the port occurred during the followup. All catheters remained patent during the month; this was related of course to the short period of the treatment. No change in the brachial-tibial index occurred in any patient. 

## 4. Discussion

PGE_1_ mechanisms of action include peripheral vasodilatation, improvement of microcirculation, and inhibition of platelet aggregation [5, 6]. Intravenously or intra-arterial infusion of PGE_1_ in patients with severe peripheral vascular disease has been well documented to be a safe and effective method of treatment in this group of patients who have a very limited—if any—choice of treatment [3, 7, 8]. Disadvantages of the intra-arterial infusion could be the presence of local side effects as rubor, swelling, and pain; on the other hand, the easiest speculated intravenous route needs a significantly increased PGE_1_ dosage (up to four times) in order to achieve the same to the arterial route effectiveness, since up to 90% of PGE_1_ undergoes metabolic degradation by the first passage from the lung parenchyma. The need of such increased dosage makes the IV treatment problematic especially in patients with borderline cardiac or renal function [3–9]; by the intra-arterial application of 20 *μ*g of PGE_1_ (a quarter of the dose required for intravenous delivery), systemic adverse effects such as hypotension (due to vasodilatation), lung edema, or cardiac failure are significantly decreased.

Using a port system, arterial access is continuous and safer compared to repeated arterial punctures. In previous reports, the risk of thrombotic complications when placed (antegrade) in small diameter target vessels (profunda or superficial femoral arteries) suffering from vascular disease was of significant concern [3, 8]. This concern was the main reason that led us to place the catheter in a retrograde route with the ipsilateral external iliac artery to be the target vessel. The rationale beyond that was that the implantation to an artery with significant flow (EIA) may contribute to longstanding patency. Infusion rate was 1 cc per minute, thus allowing the dilution to be distributed as a continuous shower to the periphery within the blood circulation [10]. Theoretically a very small portion of the total amount of PGE_1_ does not reach the extremity. Additionally we used in our protocol the maximum quantity PGE_1_ as described in the literature. These two factors contribute additively to reach maximum response and best clinical outcome.

The clinical result obtained in our patients enhances the results of previous studies for good clinical short success. Clinically important relief from rest pain was achieved in all of the patients. The two patients who had moderate response were patients with Buergers' disease who continued to smoke during treatment. Smoking may reduce the effectiveness of that type of treatment in this specific subgroup of patients.

According to the literature many different examinations exist that can objectively document changes of perfusion in the area of interest. Evaluation with transcutaneous PO_2_, laser Doppler flow, and volume flow may show improvements of microcirculation and limb perfusion and have been adequately described [11, 12]. 

In conclusion, intra-arterial infusion of PGE_1_ via a port in patients with rest pain has good short-term clinical success by creating significant peripheral vasodilatation. The placement of the catheter tip into a large and quite “healthy” artery and not near the occlusions may reduce or eliminate the risk of arterial thrombosis or spasm. We think this is an interesting new figure in this type of treatment. Further studies with more patients may be needed to document these observations.
